# Development and validation of a nomogram for early prediction of sepsis-induced coagulopathy: a multicenter study

**DOI:** 10.3389/fmed.2025.1653699

**Published:** 2025-09-09

**Authors:** Ruimin Tan, Yi Zhou, Shuwei Zhang, Jin Yang, Quansheng Du, Jingmei Wang, Yunxing Cao

**Affiliations:** ^1^Department of Critical Care, Chongqing General Hospital, Chongqing, China; ^2^Department of Critical Care, Chongqing General Hospital, Chongqing University, Chongqing, China; ^3^Department of Critical Care, Hebei General Hospital, Shijiazhuang, China; ^4^Department of Critical Care, Handan Central Hospital, Handan, China

**Keywords:** Sepsis-induced coagulopathy, pathogenic factors, nomogram, predictive model, development and validation

## Abstract

**Background:**

Sepsis-induced coagulopathy (SIC) is a vascular endothelial cell injury and coagulation disorder caused by sepsis. The aim of this study was to construct a nomogram model of the risk of early onset of SIC in patients with sepsis by analyzing the risk factors for in-hospital development of SIC.

**Methods:**

Patients with sepsis admitted to the intensive care unit (ICU) of Hebei General Hospital and Handan Central Hospital (East District) from March 1, 2021 to March 1, 2024 were retrospectively included. Sepsis patients were divided into SIC and non-SIC groups according to whether SIC occurred during hospitalization. The patient data were randomly divided into training set and testing set in the ratio of 7:3. The data of sepsis patients admitted to the ICU of Hebei General Hospital between March 1, 2024 and October 31, 2024 were then retrospectively included as the validation set for external validation. All predictors were collected within 24 h of sepsis diagnosis to enable early risk prediction. Various clinical variables were collected, and independent risk factors for early onset of SIC were screened by one-way logistic regression, least absolute shrinkage and selection operator (LASSO) regression, and multifactorial logistic and a nomogram prediction model was constructed. The model was evaluated for accuracy, goodness of fit, and clinical utility value using testing set and validation set data. The accuracy of the predictive model was assessed by using the receiver operating characteristic curve (ROC) and calculating the area under the receiver (AUC), the fit was done by calibration curve, and the clinical utility of the predictive model was assessed by decision curve analysis (DCA).

**Results:**

Among 847 patients with sepsis, SIC occurred in 480 (56.7%) patients. A nomogram model was constructed containing eight variables: lactate, oxygenation index, total protein, total bilirubin, urea, calcitoninogen, activated partial thromboplastin time, and monocyte count. In the training set, the AUC value of the model was 0.783 [95% Confidence Interval (CI): 0.746, 0.820]; in the testing set, the AUC value was 0.768 (95% CI: 0.710, 0.826); and in the validation set, the AUC value was 0.782 (95% CI: 0.708, 0.856).

**Conclusion:**

We developed a nomogram model to predict the risk of SIC in patients with sepsis and validated its potential as a clinically reliable tool. The overall accuracy and clinical utility value of the model was high and the fit was good. The nomogram model can visualize the key variables associated with SIC in sepsis patients, supporting clinicians in individualized risk assessment and guiding timely interventions to improve patient outcomes.

## 1 Introduction

Sepsis is the result of an imbalance in the host response to infection, triggering life-threatening organ function damage, and is essentially a disordered and dysregulated immune response with organ dysfunction (1). Sepsis-induced coagulopathy (SIC) is a complex pathophysiological condition triggered by sepsis, characterized by a severe disturbance of the systemic inflammatory response and coagulation system (2). According to statistics, SIC occurs in 24.0 to 60.0% of sepsis patients worldwide, and in China it is as high as 67.9%, and if not handled properly, it can develop into disseminated intravascular coagulation (DIC), which increases the mortality rate of the patients by two times (3, 4). Therefore, early identification of risk factors inducing coagulation dysfunction in sepsis patients and early warning and risk stratification of SIC patients can provide scientific basis for clinical decision-making and promote timely implementation of interventions to reduce their morbidity and mortality rates.

The concept of SIC was first proposed by the International Society for Thrombosis and Hemostasis (ISTH) in 2017, and was initially incorporated into the clinical diagnostic system as one of the key criteria for sepsis (5). SIC is essentially a complex pathophysiological sepsis-induced SIC is essentially a complex pathophysiological state induced by sepsis, and its core feature is manifested as a severe dysregulation of the systemic inflammatory response and coagulation system (2). From the viewpoint of pathogenesis, SIC involves a multifaceted pathological process of endothelial cell injury, cascade release of inflammatory mediators, and excessive activation of coagulation factors. Continued progression of these mechanisms can lead to serious complications such as DIC and multiple organ dysfunction syndrome, which significantly increase the risk of patient death (6–8). It is worth noting that there is a bidirectional synergy between inflammation and coagulation process: the exacerbation of inflammatory response can accelerate the coagulation cascade through the activation of coagulation factors; conversely, the activation of the coagulation system can further amplify the inflammatory response through the release of pro-inflammatory factors (9). This vicious circle is particularly prominent in the progression of sepsis, which can lead to extensive systemic microvascular thrombosis and ultimately to the development of DIC, which is typically characterized by hemorrhagic tendencies and microcirculatory failure (10, 11).

Nomograms are effective graphical visualization tools that help users quickly and accurately process complex data for prediction without the use of computers or other tools. Nomograms are graphical representations of complex mathematical formulas, typically using biological markers and clinical variables, and are depicted graphically, with the result being the probability of a clinical event (e.g., disease occurrence or death) for a given individual (12). In recent years, scholars have explored the role of a nomogram model constructed based on public databases in the prognosis of SIC patients and found that the model provides a better prediction of 28-day mortality in SIC patients, leading to a better assessment of prognosis (13).

This study focuses on exploring the potential value of common laboratory indicators, aiming to construct a nomogram model of early morbidity risk in SIC patients through scientific and rigorous analysis methods. This model will provide an intuitive and efficient tool for early screening of SIC patients, and help clinicians to make accurate decisions and implement timely interventions.

## 2 Materials and methods

### 2.1 Data source

A retrospective cohort of patients with sepsis admitted to the intensive care units (ICUs) of Hebei General Hospital and Handan Central Hospital (East District) between March 1, 2021, and March 1, 2024, was enrolled, provided that relatively complete clinical data were available. Sepsis patients were divided into SIC and non-SIC groups according to the occurrence of SIC during hospitalization, which was defined using the SIC score established by the ISTH. The patient data were randomly divided into training set and testing set in the ratio of 7:3. In addition, the data of sepsis patients admitted to the ICU of Hebei General Hospital between March 1, 2024 and October 31, 2024 with relatively complete information were retrospectively included as the validation set for external validation. This study complied with the review and approval criteria of the Ethics Committee of Hebei General Hospital (No. 2025-LW-0151) and the Ethics Committee of Handan Central Hospital (No. 2025112).

### 2.2 Study subjects

**Figure 1:**
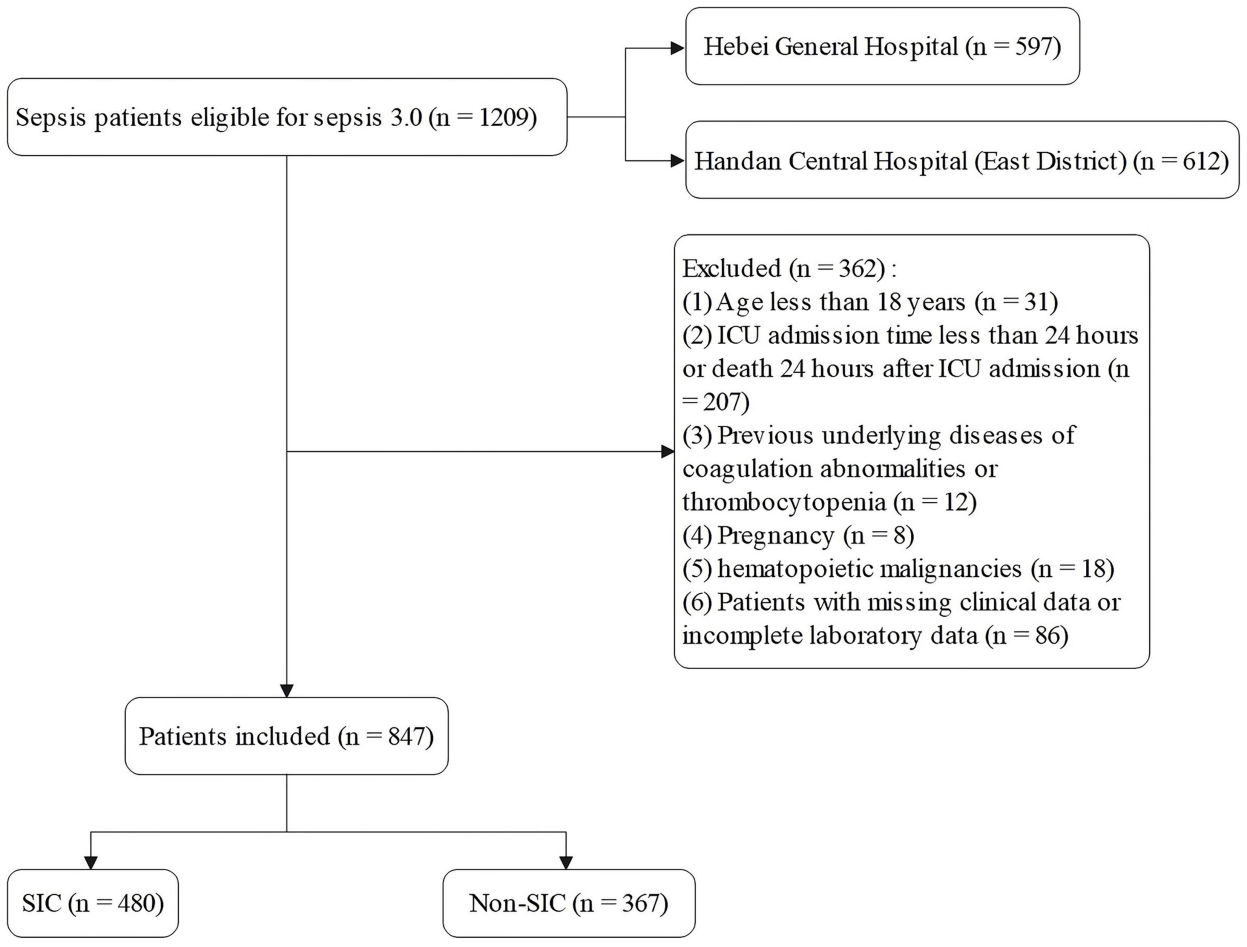
Patient screening flow.

Inclusion criteria for this study were patients who were admitted to the ICU for the first time and met the diagnostic criteria for sepsis 3.0. Exclusion criteria included (1) age <18 years; (2) ICU stay of less than 24 h or death within 24 h of admission; (3) patients with known coagulation disorders or thrombocytopenic underlying diseases (e.g., idiopathic thrombocytopenic purpura, hemophilia, severe hepatic failure); (4) patients with coagulation abnormalities present prior to ICU admission (including those associated with pregnancy, hematopoietic malignancy, history of cardiopulmonary resuscitation) or those with a sequential organ failure score (SOFA) score <2 at baseline; and (5) Cases with incomplete clinical information or laboratory data (Figure 1).

According to the criteria proposed by ISTH 2017, the diagnosis of SIC requires a comprehensive assessment of three indicators, namely Prothrombin Time-International Normalization Ratio (PT-INR), platelet count, and full SOFA score. The specific scoring rules were: PT-INR ≤ 1.2 was scored as 0 points, >1.2 was scored as 1 point, and >1.4 was scored as 2 points; platelet count ≥1.5 × 10^11^/L (150 × 10^9^/L) was scored as 0 points, <1.5 × 10^11^/L was scored as 1 point, and <1.0 × 10^11^/L (100 × 10^9^/L) was scored as 2 points; and all the SOFA scores (the sum of the scores of respiratory, cardiovascular, and hepatic systems) were scored as 2 points, renal system scores ≤ 2 were scored as 0 points, and >2 points were scored uniformly as 2 points. SIC was diagnosed if the total score of the three items was ≥4, and the sum of the individual scores of PT-INR and platelet count was >2.

### 2.3 Data extraction

In this study, clinical data were collected based on the electronic medical record systems of two healthcare organizations, and the inclusion variables contained seven dimensions: (1) demographic characteristics (gender, age); (2) underlying comorbidities (coronary heart disease, hypertension, diabetes, chronic obstructive pulmonary disease, cerebrovascular lesions, hepatic dysfunction, chronic renal disease, malignant tumors, and recent surgical history); (3) primary infection sites (respiratory system, abdominal cavity, blood, urinary system, central nervous system, skin and soft tissues, and other sites); (4) ICU rating system (Acute Physiology and Chronic Health Evaluation II [APACHE II] and SOFA scores); (5) physiological parameters (temperature, heart rate, blood pressure index [systolic blood pressure/diastolic blood pressure/mean arterial pressure], respiratory rate); (6) laboratory test index (blood gas parameters, lactate level, actual bicarbonate, oxygenation index (OI), base excess, liver and kidney function index), procalcitonin (PCT) concentration, complete set of coagulation function (prothrombin time [PT]/activated partial thromboplastin time [APTT]/fibrinogen content/thrombin time, etc.), blood parameters and electrolyte level (potassium [K]/sodium [Na]/chloride [Cl]/calcium [Ca]/phosphorus/magnesium [Mg]); and (7) clinical interventions (deep venous access establishment, mechanical ventilation implementation, anticoagulation, glucocorticosteroid application, use of vasoactive medications, continuous renal replacement therapy and albumin infusion).

All predictor variables were collected at the time when sepsis was first diagnosed according to the Sepsis-3 criteria (defined as T0). Physiological parameters and laboratory test indices were extracted within 24 h of T0. When multiple measurements were available during this window, the first recorded value was used as the baseline input. For patients who subsequently developed SIC during hospitalization, only variables collected at T0 were included; any measurements obtained after SIC onset were not used for model development or validation.

### 2.4 Statistical analysis

The normality test of the measurement data was performed using the Shapiro–Wilk method. Data that conformed to normal distribution were described by mean ± standard deviation, and comparisons between groups were made using the independent samples *t*-test; for data that did not conform to normal distribution, they were expressed as median and interquartile spacing, and comparisons between groups were made using the Wilcoxon rank-sum test for two independent samples. Count data were expressed as frequency and constitutive ratio, and differences between groups were compared using the chi-square test.

To prevent information leakage, all predictor variables were restricted to the pre-specified baseline window at the time of sepsis diagnosis (T0), and no data collected after SIC onset were included in model development or validation. To reduce the impact of multicollinearity on model stability, variables with *p* < 0.05 in univariate analysis were entered into least absolute shrinkage and selection operator (LASSO) regression. The model was penalized by introducing an L1 regularization term so that the regression coefficients of irrelevant or weakly correlated variables shrank toward zero, achieving variable screening and model simplification. The optimal penalty parameter (*λ*) was determined using 10-fold cross-validation with the minimum mean squared error criterion, thereby establishing a stable LASSO regression model and identifying variables significantly associated with SIC occurrence.

Subsequently, the variables obtained from the LASSO screening were further included in the multifactorial logistic regression analysis to clarify the independent risk factors for SIC. Based on the final regression model, a nomogram prediction model was constructed using the rms software package, and the receiver operating characteristic curve (ROC) was plotted, and the discriminative performance of the model was evaluated by calculating the area under the curve (AUC) as well as by determining the optimal cutoff values corresponding to the sensitivity and The discriminative performance of the model was evaluated by calculating AUC and determining the sensitivity and specificity indexes corresponding to the optimal cutoff value. The calibration curve was further plotted by the rms package and subjected to the Hosmer-Lemeshow goodness-of-fit test to evaluate the calibration capability of the model. The calibration of the nomogram model was assessed using calibration curves. A coordinate system was constructed with the predicted probability on the horizontal axis and the observed probability on the vertical axis. The Ideal line represents perfect agreement between predicted and observed outcomes. The Apparent line indicates the actual performance of the model on the dataset, while the Bias-corrected line reflects the prediction results obtained after 1,000 bootstrap resamples, which reduces potential overfitting and provides an internally validated estimate of model performance. The rmda software package was used to draw a decision curve analysis (DCA) plot to analyze the net benefit of the model under different threshold probabilities and determine its clinical application value. In the DCA plot, the None line represents an extreme case, i.e., the model predicts that all sepsis patients will not develop SIC, at which time the net clinical benefit is zero. The All line represents the other extreme, i.e., the model predicts that all sepsis patients will develop SIC, when the slope of the net clinical benefit is negative. The net clinical benefit is determined by the threshold probability range, i.e., the model curve lies above the None and All reference lines. If the model curve is above the None and All lines, this indicates that the model has a higher net benefit in actual clinical application. Finally, the constructed nomogram models were externally validated in an independent validation set and evaluated for their predictive performance and generalization ability.

All statistical analyses were performed in SPSS software (version: 27.0), R software (version: 4.3.1), and the difference between the two groups was considered statistically significant at *p* < 0.05.

### 2.5 Nomogram application explanation

The nomogram allows prediction of an individual’s risk of SIC by assigning points to each predictor variable. By drawing vertical lines from the value of each variable to the corresponding points axis, the individual scores are obtained. The total score, calculated by summing the points of all variables, can then be mapped to the risk-probability axis to estimate the probability of SIC occurrence. This graphical tool eliminates the need for complex calculations, enabling clinicians to rapidly and intuitively assess patient risk. Additionally, the relative contribution of each variable to the total score highlights the main risk factors, providing guidance for individualized clinical interventions and early decision-making in diagnosis and treatment.

### 2.6 Design of the validation set

To assess the applicability and stability of the model, a validation set was designed using a retrospective cohort of patients diagnosed with sepsis in the ICU of Hebei General Hospital between March 1, 2024, and October 31, 2024. The inclusion criteria were the same as before, and the final sample size of the validation set was 150 patients. The detailed process of patient selection is shown in Supplementary Figure S1.

## 3 Results

### 3.1 Baseline characteristics

A total of 847 patients with sepsis met the inclusion criteria, of whom 480 (56.7%) developed SIC. Patients were randomly assigned to a training set (*n* = 592, 336 with SIC [56.8%]) and a testing set (*n* = 255, 144 with SIC [56.5%]) in a 7:3 ratio, ensuring comparability between the two cohorts. Baseline characteristics were generally balanced between the training and testing sets, except that malignant tumors were more common in the training set (*p* = 0.04). The baseline characteristics of the two groups of patients are detailed in supplementary information Supplementary Table S1.

Within the training set, patients with SIC had a higher prevalence of hepatic insufficiency (44.94% vs. 32.81%, *p* = 0.003), elevated SOFA scores (median 9 vs. 9, *p* < 0.001), and faster heart rates (median 100 vs. 93 bpm, *p* = 0.047) compared with non-SIC patients. In terms of laboratory findings, SIC patients presented with higher lactate (2.68 vs. 1.94 mmol/L, *p* < 0.001), total bilirubin (TBIL) (21.3 vs. 14.4 μmol/L, *p* < 0.001), blood urea nitrogen (BUN) (13.69 vs. 11.5 mmol/L, *p* = 0.01), creatinine (Cr) (130.45 vs. 98.9 μmol/L, *p* < 0.001), PCT (16.59 vs. 3.9 ng/mL, *p* < 0.001), and prolonged APTT (36.9 vs. 32.3 s, *p* < 0.001). Conversely, SIC patients had lower albumin (26.7 vs. 28.65 g/L, *p* < 0.001), hemoglobin (102 vs. 109.5 g/L, *p* = 0.011), platelets (92 vs. 232.5 × 10^9^/L, *p* < 0.001), and calcium (1.96 vs. 2.02 mmol/L, *p* = 0.014). Other baseline characteristics are detailed in Table 1.

**Table 1:** Baseline characteristics of patients with and without SIC in the training set.

Variables	SIC (*n* = 336)	Non-SIC (*n* = 256)	*P*
Demographic data			
Male, *n* (%)	210 (62.50)	166 (64.84)	0.557
Age, SD	70.01 (14.73)	71.01 (14.83)	0.415
Underlying diseases, *n* (%)			
Coronary atherosclerotic heart disease	71 (21.13)	56 (21.88)	0.827
Hypertension	142 (42.26)	119 (46.48)	0.305
Diabetes	87 (25.89)	72 (28.12)	0.544
Chronic obstructive pulmonary disease	37 (11.01)	32 (12.50)	0.576
Cerebrovascular disease	114 (33.93)	102 (39.84)	0.139
Liver dysfunction	151 (44.94)	84 (32.81)	0.003
Chronic kidney disease	91 (27.08)	57 (22.27)	0.18
Malignancy	36 (10.71)	37 (14.45)	0.17
History of surgery within 3 months	158 (47.02)	127 (49.61)	0.533
Infection sites, *n* (%)			
Pulmonary	220 (65.48)	178 (69.53)	0.298
Abdominal	142 (42.26)	101 (39.45)	0.491
Blood	44 (13.10)	33 (12.89)	0.942
Urinary system	53 (15.77)	55 (21.48)	0.075
Central nervous system	4 (1.19)	9 (3.52)	0.056
Skin and soft tissue	8 (2.38)	4 (1.56)	0.484
Other	17 (5.06)	7 (2.73)	0.155
ICU disease severity scores, M (Q₁, Q₃)			
APACHE score	24.00 [19.00, 29.00]	23.00 [18.00, 28.00]	0.063
SOFA score	9.00 [7.75, 12.00]	9.00 [7.00, 10.00]	<0.001
Vital signs, M (Q₁, Q₃)			
Temperature (°C)	36.50 [36.00, 37.20]	36.50 [36.00, 37.00]	0.137
HR (times/min)	100.00 [84.00, 113.00]	93.00 [82.00, 113.00]	0.047
SBP (mmHg)	121.00 [105.00, 140.00]	121.00 [105.00, 138.25]	0.997
DBP (mmHg)	66.00 [56.00, 76.00]	67.00 [57.00, 78.00]	0.308
MAP (mmHg)	85.00 [75.17, 94.67]	85.83 [75.92, 94.67]	0.601
RR (times/min)	21.00 [16.00, 28.00]	20.00 [16.00, 25.00]	0.281
Laboratory tests, M (Q₁, Q₃)			
pH	7.36 [7.29, 7.42]	7.39 [7.31, 7.44]	0.004
Lactate (mmol/L)	2.68 [1.70, 4.70]	1.94 [1.40, 3.10]	<0.001
AB (mmol/L)	20.25 [16.50, 23.70]	22.00 [18.50, 25.52]	<0.001
OI	202.50 [135.12, 281.82]	245.20 [166.65, 354.35]	<0.001
BE (mmol/L)	−4.85 [−8.00, −1.50]	−2.88 [−6.30, 0.64]	<0.001
TP (g/L)	48.85 [42.90, 55.12]	53.90 [47.08, 61.28]	<0.001
ALB (g/L)(μmol/L)	26.70 [23.17, 30.92]	28.65 [24.98, 32.60]	<0.001
TBIL (μmol/L)	21.30 [12.50, 38.17]	14.40 [9.50, 21.83]	<0.001
DBIL (μmol/L)	9.55 [5.20, 20.45]	5.40 [3.20, 10.40]	<0.001
ALT (U/L)	32.10 [16.00, 78.93]	24.00 [13.00, 49.07]	0.001
AST (U/L)	61.50 [31.88, 134.73]	33.00 [21.00, 70.25]	<0.001
K (mmol/L)	4.00 [3.60, 4.50]	4.15 [3.70, 4.68]	0.038
Na (mmol/L)	140.40 [135.45, 145.00]	138.00 [135.00, 142.57]	0.014
Cl (mmol/L)	106.00 [101.60, 111.00]	104.00 [99.00, 108.28]	<0.001
Ca (mmol/L)	1.96 [1.83, 2.16]	2.02 [1.89, 2.16]	0.014
PO_4_ (mmol/L)	1.14 [0.80, 1.51]	1.14 [0.82, 1.40]	0.72
Mg (mmol/L)	0.82 [0.70, 0.92]	0.86 [0.73, 0.99]	0.004
BUN (mmol/L)	13.69 [8.72, 21.33]	11.50 [7.24, 19.33]	0.01
Cr (μmol/L)	130.45 [81.80, 205.22]	98.90 [63.02, 158.32]	<0.001
PCT (ng/mL)	16.59 [2.90, 59.87]	3.90 [0.86, 26.38]	<0.001
PT (s)	16.10 [14.20, 18.10]	13.70 [12.40, 15.20]	<0.001
PT-INR	1.45 [1.31, 1.60]	1.16 [1.06, 1.30]	<0.001
APTT (s)	36.90 [32.70, 44.90]	32.30 [28.80, 36.62]	<0.001
FIB (g/L)	5.23 [3.42, 12.70]	5.91 [4.16, 11.92]	0.09
TT (s)	15.60 [5.63, 17.30]	15.40 [6.06, 17.20]	0.817
WBC (10^9^/L)	11.26 [5.95, 17.57]	12.95 [9.10, 18.56]	0.002
NEU (10^9^/L)	9.63 [4.98, 16.46]	11.46 [7.36, 16.64]	0.012
LYM (10^9^/L)	0.52 [0.32, 0.82]	0.80 [0.46, 1.30]	<0.001
MON (10^9^/L)	0.31 [0.14, 0.51]	0.47 [0.26, 0.84]	<0.001
Hb (g/L)	102.00 [88.00, 123.00]	109.50 [91.00, 128.00]	0.011
RDW (fL)	48.30 [45.00, 54.00]	48.70 [45.10, 52.85]	0.64
PLT (10^9^/L)	92.00 [52.00, 125.25]	232.50 [189.00, 299.50]	<0.001
Interventions, *n* (%)			
Deep venous catheterization	279 (83.04)	190 (74.22)	0.009
Anticoagulant drugs	147 (43.75)	137 (53.52)	0.018
Mechanical ventilation	295 (87.80)	231 (90.23)	0.351
Hormones	240 (71.43)	185 (72.27)	0.823
Vasoactive drugs	319 (94.94)	247 (96.48)	0.364
CRRT	188 (55.95)	139 (54.30)	0.688
Infusion of human albumin	256 (76.19)	177 (69.14)	0.055

Z, Mann–Whitney test, χ^2^, Chi-square test. M, Median, Q₁, 1st Quartile, Q₃, 3st Quartile. SIC, sepsis-induced coagulopathy; APACHE, acute physiology and chronic health evaluation II; SOFA, sequential organ failure assessment; HR, heart rate; SBP, systolic blood pressure; DBP, diastolic blood pressure; MAP, mean arterial pressure; RR, respiratory rate; AB, actual bicarbonate; OI, oxygenation index; BE, base excess; TP, total protein; ALB, albumin; TBIL, total bilirubin; DBIL, direct bilirubin; ALT, alanine aminotransferase; AST, aspartate aminotransferase; K, potassium; Na, sodium; Cl, chloride; Ca, calcium; PO_4_, phosphorus; Mg, magnesium; BUN, blood urea nitrogen; Cr, creatinine; PCT, procalcitonin; PT, prothrombin time; PT-INR, prothrombin time-international normalization ratio; APTT, activated partial thromboplastin time; FIB, fibrinogen; TT, thrombin time, WBC, white blood cells; NEU, neutrophils; LYM, lymphocytes; MON, monocytes; Hb, hemoglobin; RDW, red blood cell distribution width; PLT, platelet.

### 3.2 Feature selection and establishment of a nomogram model

**Figure 2:**
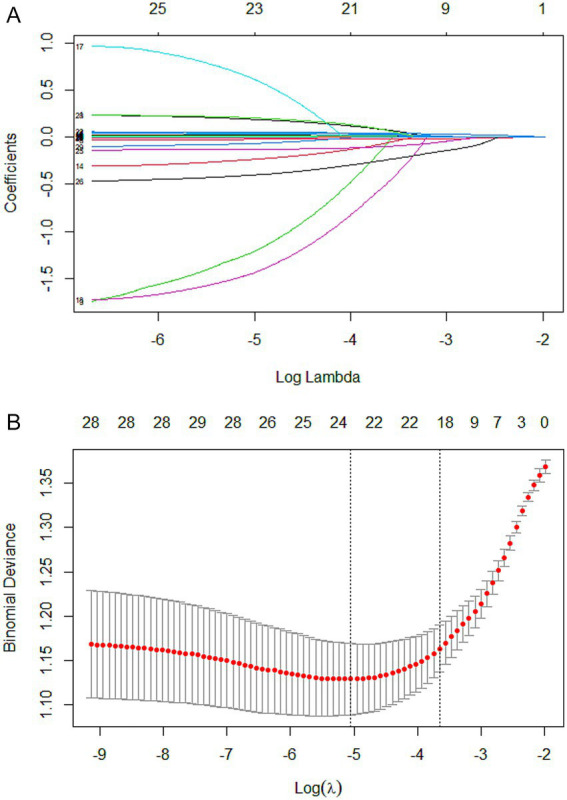
Lasso regression-based variable screening. **(A)** Variation characteristics of variable coefficients. Each colored line represents the trajectory of a specific variable’s coefficient (total of 33 variables) across different values of the regularization parameter (log *λ*). As λ decreases (from left to right), more variables are included in the model, and their coefficients shift from zero toward their ordinary least squares estimates. **(B)** The process of selecting the optimal value of the parameter λ in the lasso regression model is carried out by the cross-validation method. The red dotted line indicates the optimal λ value selected via cross-validation (lambda.min), which corresponds to the model achieving the minimum mean cross-validated error. The upper x-axis shows the log(λ) values, while the lower x-axis indicates the corresponding number of nonzero coefficients in the model at each λ.

In the training set, candidate variables were first initially screened using one-way logistic regression analysis, and a total of 33 variables were included in the analysis, all of which were statistically significant (*p* < 0.05) in the one-way analysis. Subsequently, LASSO regression was used to further compress the variables, and the optimal penalty parameter *λ* was determined by combining the 10-fold cross-validation (CV), and the lowest mean square error was used as a criterion for feature selection. The dynamics of variable coefficient changes during the screening process of LASSO regression is shown in Figure 2A, while Figure 2B demonstrates the process of selecting the optimal value of *λ* in the cross-validation. In the end, the LASSO regression screened a total of 19 variables that were closely related to the occurrence of SIC, namely: hepatic insufficiency, heart rate, pH, blood lactate, OI, total protein (TP), TBIL, aspartate aminotransferase, K, Cl, Mg, BUN, Cr, PCT, APTT, lymphocyte count, monocyte count, hemoglobin, and deep intravenous catheterization.

**Figure 3:**
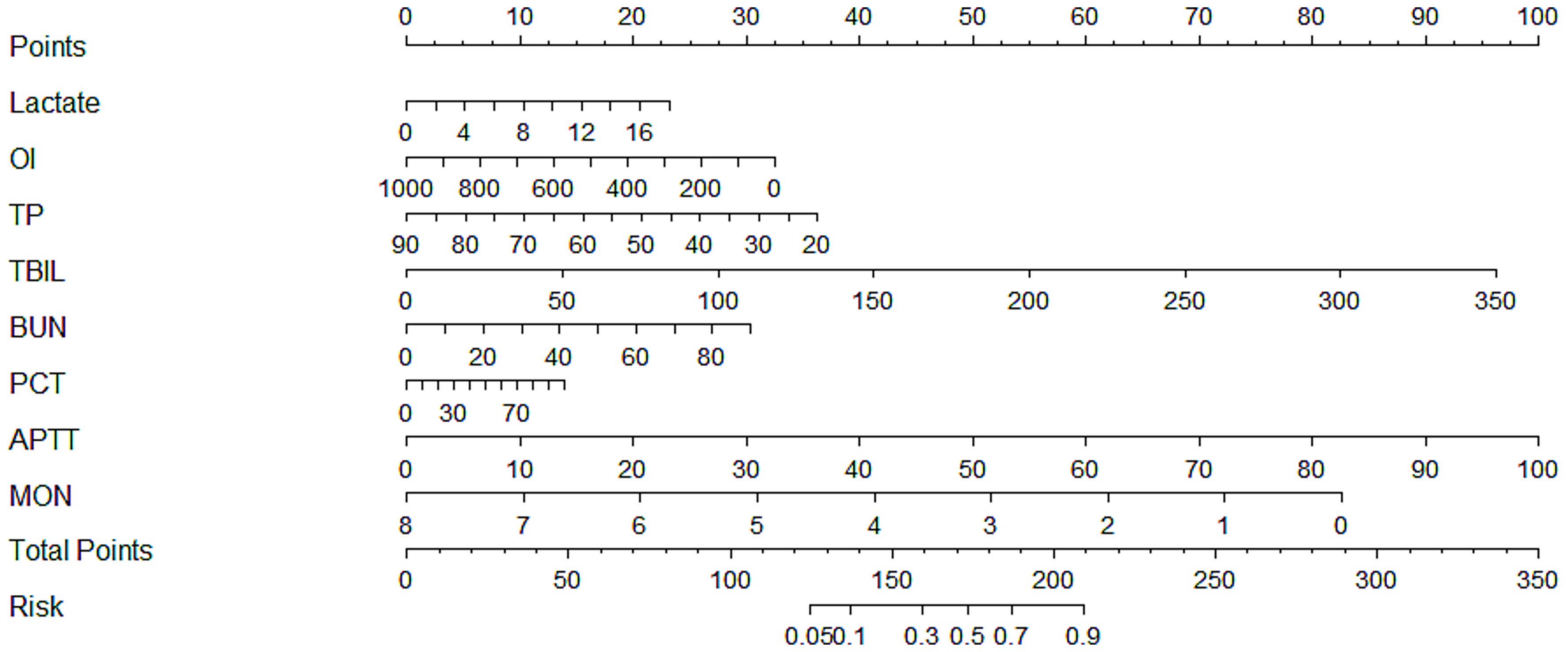
A nomogram model of morbidity in SIC patients. SIC, sepsis-induced coagulopathy; OI, oxygenation index; TP, total protein; TBIL, total bilirubin; BUN, blood urea nitrogen; PCT, procalcitonin; APTT, activated partial thromboplastin time; MON, monocytes.

Based on the above variables screened by LASSO regression, further multifactorial logistic regression analysis was incorporated to identify the risk factors independently associated with the risk of SIC development, and to construct a risk prediction model with strong predictive ability and clinical utility. Multivariate logistic regression identified eight independent predictors of SIC: lactate (odds ratio [OR] = 1.081, 95% CI: 1.002–1.167, *p* = 0.044), OI (OR = 0.998, 95% CI: 0.997–0.999, *p* = 0.009), TP (OR = 0.969, 95% CI: 0.951–0.987, *p* = 0.001), TBIL (OR = 1.017, 95% CI: 1.008–1.025, *p* < 0.001), BUN (OR = 1.021, 95% CI: 1.005–1.036, *p* = 0.009), PCT (OR = 1.008, 95% CI: 1.002–1.015, *p* = 0.008), APTT (OR = 1.063, 95% CI: 1.040–1.086, *p* < 0.001), and monocyte count (OR = 0.534, 95% CI: 0.364–0.783, *p* = 0.001). Detailed results are provided in Table 2. Based on the previously screened independent predictors of SIC occurrence, this study further constructed a nomogram model (Figure 3), aiming to visualize the complex regression formula graphically.

**Table 2:** Multivariate logistic regression analysis of factors associated with the incidence of SIC.

Variables	*β*	SE	Wald	*P*	OR (95% CI)
Lactate	0.078	0.039	4.072	0.044	1.081 (1.002–1.167)
OI	−0.002	0.001	6.881	0.009	0.998 (0.997–0.999)
TP	−0.032	0.01	10.757	0.001	0.969 (0.951–0.987)
TBIL	0.017	0.004	15.548	<0.001	1.017 (1.008–1.025)
BUN	0.021	0.008	6.916	0.009	1.021 (1.005–1.036)
PCT	0.008	0.003	7.003	0.008	1.008 (1.002–1.015)
APTT	0.061	0.011	29.657	<0.001	1.063 (1.040–1.086)
MON	−0.628	0.195	10.338	0.001	0.534 (0.364–0.783)
Constant	−0.702	0.673	1.089	0.297	

*β*, partial regression coefficient; SE, standard error; OR, odds ratio; CI, confidence interval. SIC, sepsis-induced coagulopathy; OI, oxygenation index; TP, total protein; TBIL, total bilirubin; BUN, blood urea nitrogen; PCT, procalcitonin; APTT, activated partial thromboplastin time; MON, monocytes.

### 3.3 Validation and evaluation of model effectiveness

#### 3.3.1 Validation set

During the study period, a total of 184 patients with sepsis were initially identified. Based on the pre-specified inclusion and exclusion criteria, 25 patients with an ICU stay of less than 24 h, 1 patient with pre-existing hematologic disorders such as thrombocytopenia, 3 patients with co-morbid hematopoietic malignancies, and 5 patients with incomplete laboratory data were excluded. Ultimately, 150 patients were included in the validation set for this study (Supplementary Figure S1). The incidence of SIC in this cohort was 56%. The baseline characteristics of relevant predictor variables in the validation set are summarized in Supplementary Table S2.

#### 3.3.2 ROC curves and AUC values

**Figure 4:**
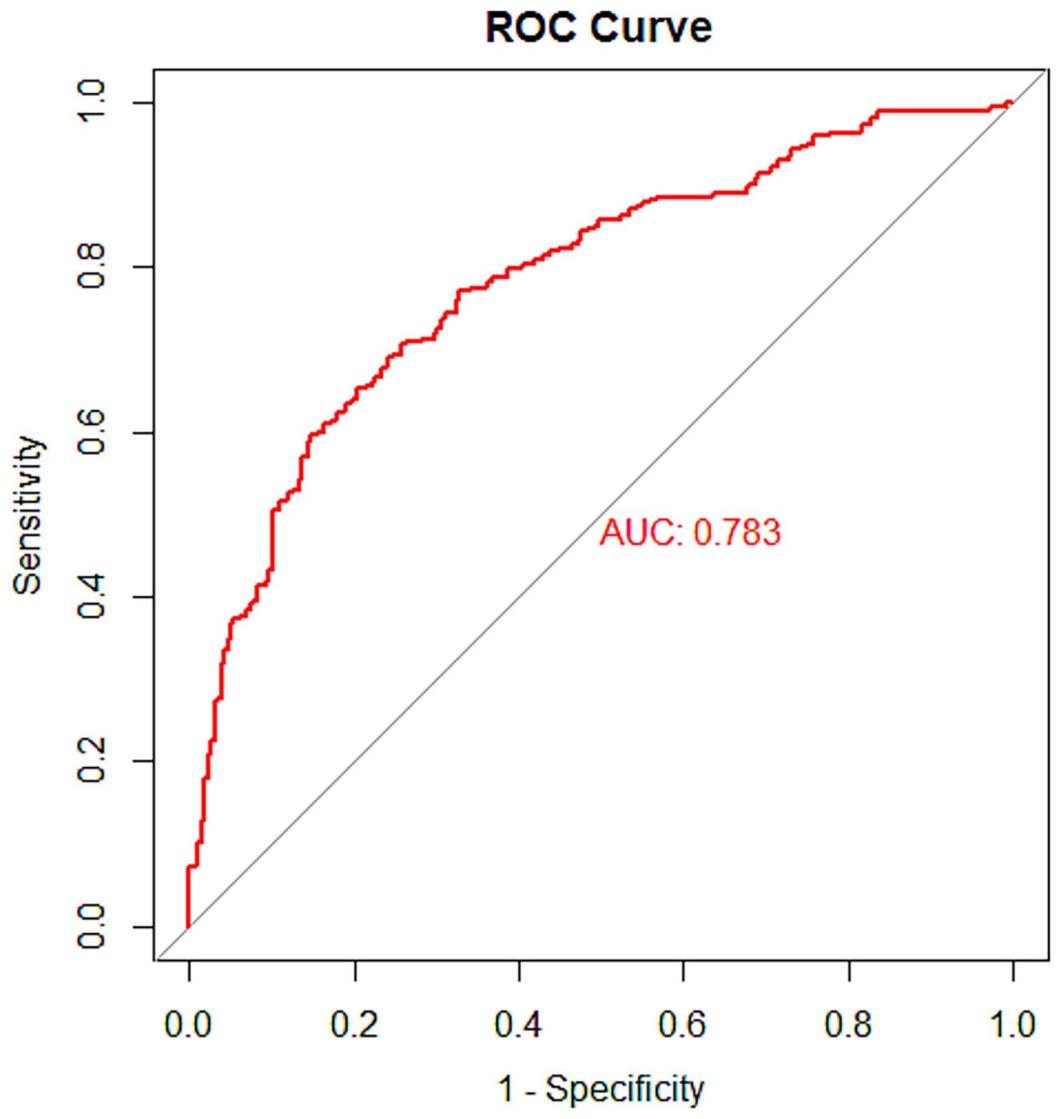
ROC curve of the nomogram model in the training set.

In the training set, the nomogram model achieved an AUC of 0.783 (95% CI: 0.746–0.820). In the testing set, the model yielded an AUC of 0.768 (95% CI: 0.710–0.826). In the validation set, the AUC was 0.782 (95% CI: 0.708–0.856). These results indicate that the nomogram model exhibited good predictive performance for the risk of early-onset SIC. The ROC curves of the model in the training, testing, and validation sets are shown in Figure 4, Supplementary Figures S2A,B, respectively, providing a visual representation of model performance. Detailed results, including AUC values, 95% CI, sensitivity, specificity, positive predictive value (PPV), and negative predictive value (NPV) for each dataset, are presented in Table 3.

**Table 3:** Detailed results of the nomogram model for each dataset.

Dataset	AUC	95% CI	Sensitivity	Specificity	PPV	NPV
Training set	0.783	0.746–0.820	0.652	0.797	0.808	0.636
Testing set	0.768	0.710–0.826	0.632	0.847	0.843	0.562
Validation set	0.782	0.708–0.856	0.869	0.576	0.723	0.776

AUC, area under the curve; CI, confidence interval; PPV, positive predictive value; NPV, negative predictive value.

#### 3.3.3 Calibration graph, brier score, and calibration error

**Figure 5:**
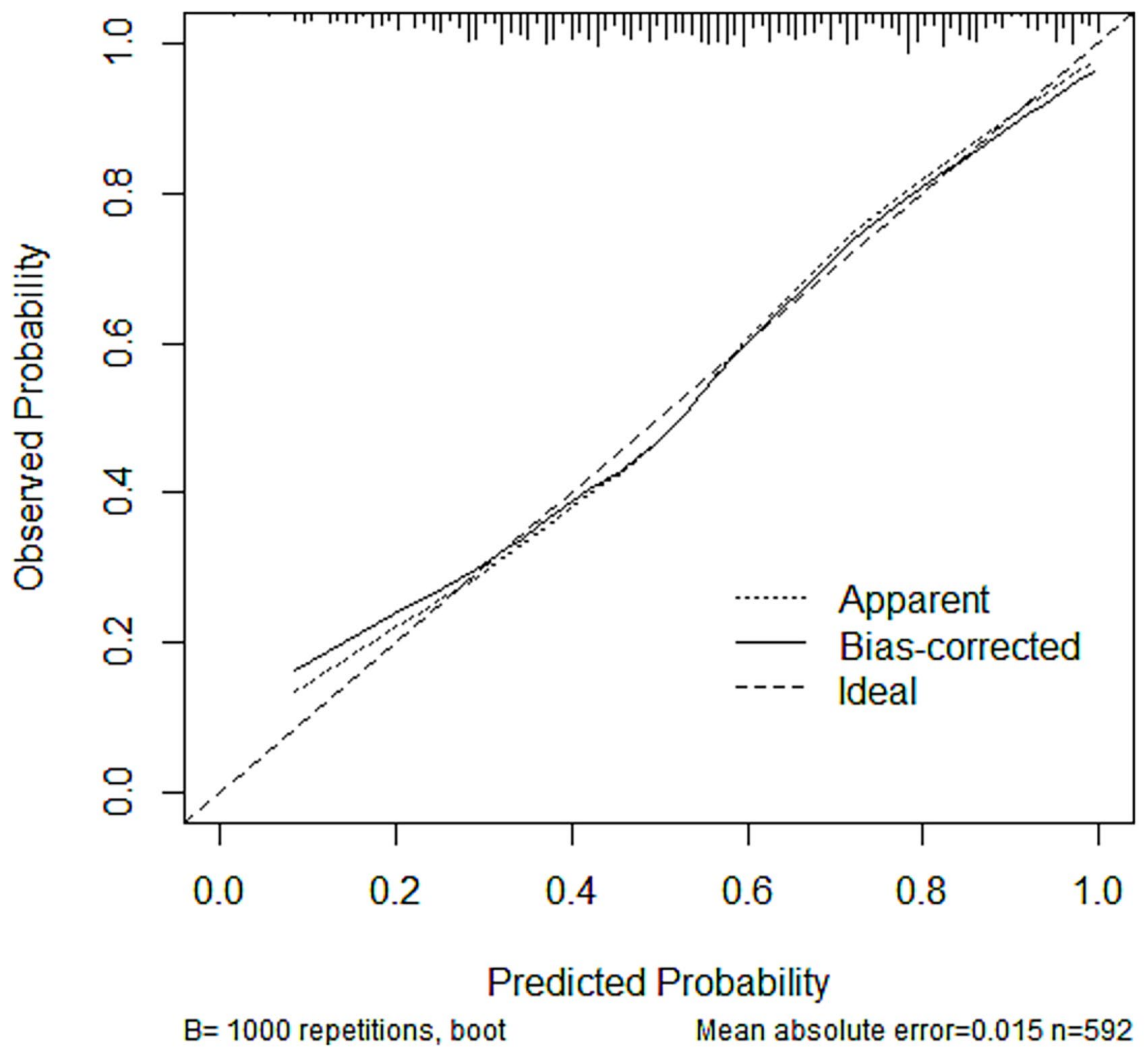
Calibration curves of the nomogram model in the training set.

The calibration analysis showed that in all datasets, the Bias-corrected line was highly consistent with the Apparent line, with only minor deviations, indicating that the model exhibited good reliability and stability. The calibration curves for the training, testing, and validation sets are presented in Figure 5, Supplementary Figures S3A,B, respectively. In addition, the Brier score and calibration error were calculated to further evaluate the calibration performance of the model in each dataset, as summarized in Table 4.

**Table 4:** Evaluation of the calibration of the nomogram model across datasets.

Dataset	Brier scores	Calibration errors
Training set	0.188	0.015
Testing set	0.195	0.027
Validation set	0.198	0.033

#### 3.3.4 DCA curves

**Figure 6:**
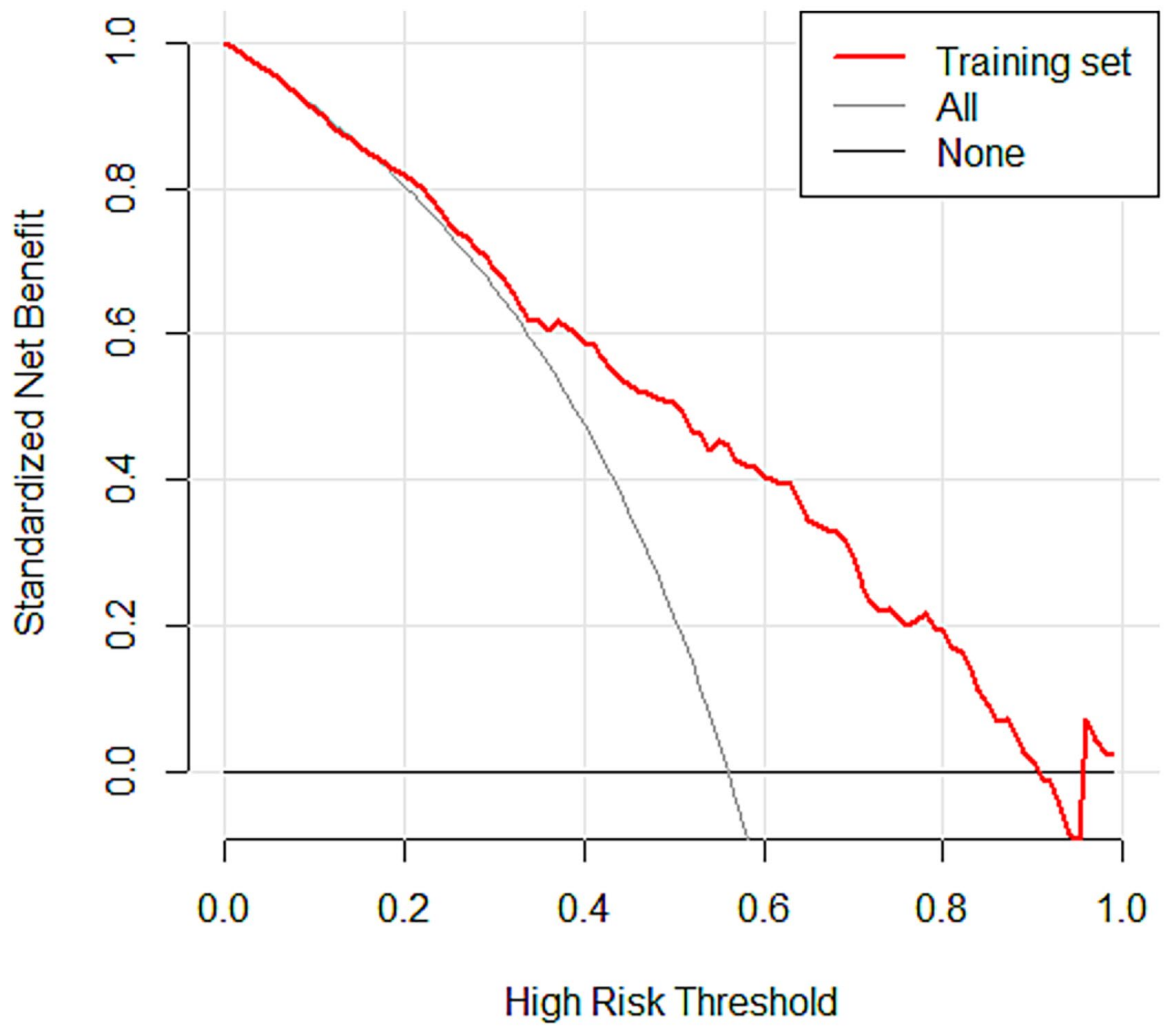
DCA curves of the nomogram model in the training set.

DCA was performed to evaluate the clinical utility of the prediction model in the training, testing, and validation sets (Figure 6; Supplementary Figures S4A,B). In the DCA plots, the horizontal axis represents the threshold probability, and the vertical axis represents the net benefit. The results showed that the nomogram model provided higher net benefit than the treat-all and treat-none strategies across most clinically relevant threshold ranges. In particular, within the threshold range of 0.1 to 0.8, the model curve remained consistently above the reference strategies, suggesting favorable clinical applicability. Furthermore, the model maintained a comparable net benefit in the external validation set, supporting its generalizability and robustness across different datasets.

## 4 Discussion

In this study, we constructed a nomogram model to predict the risk of SIC in patients with sepsis using multicenter real-world clinical data. The model’s performance was evaluated across multiple dimensions, including predictive accuracy, calibration, and decision curve analysis. The validation set results indicated consistent predictive performance, with the model achieving satisfactory AUC values, reliable calibration, and favorable net benefit across clinically relevant threshold probabilities. These findings suggest that the nomogram may assist clinicians in identifying patients at higher risk of SIC and support individualized decision-making in early intervention.

Coagulation changes in sepsis patients are dynamic, beginning with early coagulation abnormalities, which may progress to SIC and eventually DIC (14). Coagulation disorders are reported in approximately 50–70% of sepsis patients, with nearly 35% developing secondary DIC (15, 16). Coagulation dysfunction is nearly universal in sepsis and is strongly associated with multi-organ failure and increased mortality (17, 18). In SIC, pathogens and inflammatory mediators trigger thrombus formation through mechanisms including upregulation of procoagulant pathways, downregulation of physiological anticoagulant systems, and inhibition of fibrinolysis (19). Thrombin, a central regulator, binds to protease-activated receptor-1 on monocytes and neutrophils, amplifying pro-inflammatory and pro-coagulant responses (20, 21). Simultaneously, inflammatory cytokine release promotes neutrophil adhesion to endothelial cells, activating complement and coagulation cascades (22). Complement C5a further induces the release of damage-associated molecular patterns and pathogen-associated molecular patterns, exacerbating coagulation dysfunction and inflammation (23). Monocytes and macrophages detect and phagocytose pathogens, express pattern recognition receptors (e.g., Toll-like and Fcγ receptors), and release inflammatory mediators, perpetuating cycles of inflammation and coagulation (24–26). Given the central role of the inflammatory–coagulation feedback loop and endothelial injury in accelerating SIC progression, early identification is therefore critical to guide timely intervention and targeted therapies.

Current approaches for predicting the risk of SIC include sensitive serum biomarkers and clinical scoring systems. Biomarkers such as PCT and lactate are readily obtained but provide only a snapshot of the patient’s physiological state, with limited sensitivity and specificity for predicting SIC (27–29). Clinical scores, including the SOFA, rapid sequence organ failure, and systemic inflammatory response syndrome (SIRS) scores, are straightforward but suffer from limited timeliness and accuracy and are influenced by multiple pathophysiologic factors (30, 31). Recent studies have highlighted the prognostic value of composite biomarkers in reflecting systemic inflammation, nutritional status, and immune function in sepsis patients. For example, Sarıdaş and Çetinkaya demonstrated that the CALLY index, a composite biomarker reflecting inflammation, nutrition, and immunity, has significant prognostic relevance in sepsis, supporting the use of integrated biomarker-based risk assessment (32). Additionally, Saridas et al. illustrated the clinical utility of novel inflammatory markers in predicting complicated appendicitis, emphasizing the broader applicability of composite biomarkers in acute inflammatory conditions (33). These findings underscore the potential value of incorporating multiple clinical and laboratory parameters into predictive models for early risk stratification in sepsis. Overall, existing methods are limited by low predictive strength, instability, and procedural complexity. Developing efficient and individualized prediction models is thus a priority. Nomograms integrate multiple variables into a single, user-friendly tool, facilitating quantitative risk assessment and clinical decision-making. In this study, we constructed a nomogram for early morbidity risk in SIC, supporting early warning, risk stratification, and intervention.

Eight independent risk factors—lactate, OI, TP, TBIL, BUN, PCT, APTT, and monocyte count—were identified and incorporated into the predictive model.

Lactate reflects tissue perfusion and oxygen metabolism and is widely used to detect microcirculatory disturbances in sepsis (34). Impaired microcirculation and hepatic or renal dysfunction reduce lactate clearance, causing hyperlactatemia (35). Elevated lactate damages endothelial cells, alters permeability, triggers exogenous coagulation, and promotes microthrombus formation, creating a vicious cycle of hypoperfusion and coagulation dysfunction (36–38). In this study, lactate levels were significantly higher in SIC patients (OR = 1.018, 95%CI: 1.002 ~ 1.167, *p* = 0.044), confirming its role as an independent risk factor. Monitoring lactate may facilitate early identification of patients at high risk for SIC, provide insights into potential coagulation dysfunction and tissue hypoxia, and offer guidance for timely fluid resuscitation, thus supporting improved clinical management.

OI, the ratio of arterial oxygen partial pressure to inspired oxygen fraction, reflects pulmonary gas exchange. Sepsis-induced acute lung injury or acute respiratory distress syndrome (ARDS) impairs OI through alveolar exudation, reduced compliance, and microvascular thrombosis (39–41). SIC-related coagulation dysfunction contributes to reduced OI, further exacerbating lung injury. In our cohort, SIC patients exhibited significantly lower OI (OR = 0.998, 95%CI: 0.997–0.999, *p* = 0.009), highlighting its value as an early risk predictor for SIC. Monitoring OI informs pulmonary function assessment and prognosis evaluation.

TP encompasses all serum proteins, including albumin and globulins, essential for colloid osmotic pressure, transport, and immune function. Hepatic dysfunction in sepsis reduces TP and coagulation factor synthesis, promoting SIC. Hypoproteinemia activates coagulation and inflammation, disrupting anticoagulant homeostasis (42, 43). TP was lower in SIC patients (OR = 0.969, 95%CI: 0.951 ~ 0.987, *p* = 0.001), suggesting a poor prognosis. Correction of hypoproteinemia may improve coagulation and survival outcomes.

TBIL, the sum of direct and indirect bilirubin, rises with hepatic dysfunction and SIRS-induced cholestasis (44, 45). Elevated TBIL reflects oxidative stress, endothelial injury, and coagulation activation, contributing to microcirculatory disturbances. TBIL was significantly higher in SIC patients (OR = 1.017, 95%CI: 1.008 ~ 1.025, *p* < 0.001), serving as a marker of liver injury and coagulation impairment. Monitoring TBIL guides therapeutic strategies to reduce inflammation and oxidative stress.

BUN, an end product of protein metabolism, accumulates due to renal impairment and hypercatabolism in SIC (46, 47). Elevated BUN worsens coagulation by reducing clearance of coagulation factors and fibrin degradation products, increasing blood viscosity and anemia. SIC patients had higher BUN (OR = 1.021, 95%CI: 1.005 ~ 1.036, *p* = 0.009). BUN monitoring informs renal perfusion strategies and inflammatory control.

PCT, a precursor of calcitonin, markedly elevates under inflammatory conditions. In SIC, PCT stimulates von Willebrand factor release and microthrombosis, exacerbating coagulation disorders (48). Higher PCT was observed in SIC patients (OR = 1.008, 95%CI: 1.002 ~ 1.015, *p* = 0.008). Combining PCT with coagulation tests enables comprehensive assessment and guides targeted anti-infective and anticoagulant therapy.

APTT evaluates endogenous coagulation. In SIC, inflammatory mediators activate coagulation pathways, consuming factors and prolonging APTT. Dysfunctional anticoagulant systems, including protein C and antithrombin, further disrupt hemostasis (49). SIC patients exhibited prolonged APTT (OR = 1.063, 95%CI: 1.040 ~ 1.086, *p* < 0.001), indicating factor depletion and fibrinolytic activation. APTT informs decisions on coagulation factor supplementation or anticoagulation therapy.

Monocytes regulate immune response and inflammation. In SIC, myelosuppression reduces monocyte counts, impairing pathogen clearance and cytokine production, perpetuating coagulation activation (50). Lower monocyte counts in SIC patients (OR = 0.534, 95%CI: 0.364 ~ 0.783, *p* = 0.001) reflect severe immune dysfunction and poor prognosis.

This study has several advantages. (1) Multicenter data and rigorous methodological design: Based on multicenter data from Hebei General Hospital and Handan Central Hospital (East District), this study included 847 patients with sepsis and was randomly divided into training and testing sets using a 7:3 ratio, along with independent external validation (150 cases). The study strictly adhered to the TRIPOD statement, and variables were screened by univariate analysis, LASSO regression, and multifactorial logistic regression to ensure the scientific validity and stability of the model. (2) Construction and validation of the integrated predictive model: This study integrated eight key clinical indicators (lactate, OI, TP, TBIL, BUN, PCT, APTT, and monocyte count), constructed a nomogram model, and demonstrated good discriminative ability in the training set (AUC = 0.783), testing set (AUC = 0.768), and validation set (AUC = 0.782), suggesting that the model has high prediction accuracy. (3) Multi-dimensional model evaluation: This study not only assessed the discriminative ability of the model through ROC curves, but also evaluated the calibration and clinical utility of the model by combining calibration curves (Brier score, calibration error) and DCA curves. The DCA results showed that the model had a significant net benefit within the risk threshold range of 0.1–0.8, which supports its application value in clinical decision making. (4) Clinical applicability: The nomograms are presented in a visual way, which facilitates clinicians to quickly assess the risk of SIC in patients without complex calculations. In addition, the indicators included in the study are all routine laboratory tests (e.g., lactate, PCT, APTT, etc.), which are easy to generalize in clinical practice. (5) Mechanistic explanation and clinical significance are clear: This study discussed in detail the association of each predictor variable (e.g., lactate, oxygenation index, monocyte count) with the pathogenesis of SIC, provided theoretical support for the biological plausibility of the model, and suggested targeted clinical interventions (e.g., monitoring of lactate, correction of hypoproteinemia, etc.).

In order to provide a practical guide for clinicians on the use of our nomogram, we present a representative clinical scenario. Consider a sepsis patient presenting with a lactate level of 4 mmol/L, a monocyte count of 0.3 × 10^9^/L, and an APTT of 50 s. By applying our nomogram, the total point score for this patient is calculated as 120, corresponding to an estimated risk of developing SIC of approximately 75%. This high predicted risk would suggest that the attending physician should implement closer monitoring of coagulation parameters, consider early anticoagulant therapy if indicated, and evaluate the need for ICU admission. Such an example illustrates how different clinical variables interact within the nomogram to generate a quantitative risk assessment, thereby facilitating timely and evidence-based clinical decision-making. By providing clinicians with a clear, individualized risk estimate, the nomogram can support rapid identification of high-risk patients, optimize resource allocation, and potentially improve patient outcomes. We anticipate that integrating this tool into daily clinical practice will enhance the precision of SIC management and increase clinician confidence in initiating appropriate interventions.

This study has several limitations. First, as a retrospective study, data were obtained from electronic medical records, which may contain incomplete or inaccurate entries, potentially leaving some confounding factors unaddressed. Second, despite including multiple clinical variables, certain factors such as genetic or environmental influences were not captured, which may limit the model’s predictive ability for all sepsis patients. Third, the analysis focused on baseline parameters at ICU admission and did not fully account for dynamic changes in coagulation function, which may restrict risk prediction over time. Fourth, traditional logistic regression was used, without exploring machine learning or deep learning approaches that could capture nonlinear relationships and potentially improve predictive accuracy. Fifth, although a multicenter study was conducted, all patients were from hospitals in Hebei Province, which limits generalizability to other regions with different disease spectra, medical resources, or environmental conditions. Additionally, two specific limitations were considered. Sixth, the overall proportion of missing values was relatively low (<5%), and records with missing data were excluded rather than imputed. While this approach is unlikely to substantially affect results, different strategies for handling missing data could influence model stability and performance. Seventh, although data were collected on the day of sepsis diagnosis, subsequent treatments—such as anticoagulants, corticosteroids, or vasopressors—may have affected the development of SIC, representing a potential confounding factor that our model cannot fully account for. Future prospective studies incorporating dynamic treatment data could further improve predictive accuracy and clinical applicability. These limitations highlight the need for cautious interpretation of our findings and underscore the importance of further validation in broader, dynamically monitored patient populations.

## 5 Conclusion

We developed a nomogram model to predict the risk of SIC in patients with sepsis and validated its potential as a clinically reliable tool. The overall accuracy and clinical utility value of the model was high and the fit was good. The nomogram model can visualize the main causes of SIC in sepsis patients to precisely guide clinicians to take individualized diagnostic and therapeutic measures to reduce the incidence of poor prognosis in sepsis patients.

## Data Availability

The data analyzed in this study is subject to the following licenses/restrictions: the dataset used in this study is not publicly available due to patient privacy concerns and institutional regulations. However, the data are available from the corresponding author upon reasonable request and with appropriate ethical approval. Requests to access these datasets should be directed to YC, 104387251@qq.com.
