# Supplementary material for: Development and validation of a nomogram for early prediction of sepsis-induced coagulopathy: a multicenter study
Source: Front Med (Lausanne). 2025 Sep 9;12:1653699. doi: 10.3389/fmed.2025.1653699 (PMC12454385; doi:10.3389/fmed.2025.1653699)

**Supplementary Table**

Table S1. Comparison of baseline characteristics between the training and testing sets

| Variables | training set (n=592) | testing set (n=255) | *P* |
| --- | --- | --- | --- |
| Demographic data |  |  |  |
| Male, n(%) | 376 (63.51) | 149 (58.43) | 0.162 |
| Age, SD | 70.44 (14.77) | 71.80 (15.57) | 0.228 |
| Underlying diseases, n(%) |  |  |  |
| Coronary atherosclerotic heart disease | 127 (21.45) | 61 (23.92) | 0.428 |
| Hypertension | 261 (44.09) | 112 (43.92) | 0.964 |
| Diabetes | 159 (26.86) | 78 (30.59) | 0.267 |
| Chronic obstructive pulmonary disease | 69 (11.66) | 38 (14.90) | 0.192 |
| Cerebrovascular disease | 216 (36.49) | 95 (37.25) | 0.831 |
| Liver dysfunction | 235 (39.70) | 86 (33.73) | 0.100 |
| Chronic kidney disease | 148 (25.00) | 66 (25.88) | 0.786 |
| Malignancy | 73 (12.33) | 45 (17.65) | 0.04 |
| History of surgery within 3 months | 285 (48.14) | 127 (49.80) | 0.657 |
| Infection sites, n(%) |  |  |  |
| Pulmonary | 398 (67.23) | 178 (69.80) | 0.461 |
| Abdominal | 243 (41.05) | 102 (40.00) | 0.776 |
| Blood | 77 (13.01) | 33 (12.94) | 0.979 |
| Urinary system | 108 (18.24) | 44 (17.25) | 0.731 |
| Central nervous system | 13 (2.20) | 4 (1.57) | 0.550 |
| Skin and soft tissue | 12 (2.03) | 6 (2.35) | 0.763 |
| Other | 24 (4.05) | 8 (3.14) | 0.521 |
| ICU disease severity scores, M (Q₁, Q₃) |  |  |  |
| APACHE score | 23.00 [18.00, 28.00] | 23.00 [17.00, 28.00] | 0.124 |
| SOFA score | 9.00 [7.00, 11.00] | 9.00 [6.00, 11.00] | 0.226 |
| Vital signs, M (Q₁, Q₃) |  |  |  |
| Temperature(℃) | 36.50 [36.00, 37.10] | 36.50 [36.00, 37.10] | 0.988 |
| HR(times/min) | 96.50 [83.00, 113.00] | 98.00 [83.50, 115.50] | 0.379 |
| SBP(mmHg) | 121.00 [105.00, 140.00] | 122.00 [107.00, 142.00] | 0.583 |
| DBP(mmHg) | 67.00 [56.00, 77.25] | 67.00 [57.00, 78.00] | 0.655 |
| MAP(mmHg) | 85.67 [75.33, 94.67] | 84.67 [75.83, 95.67] | 0.790 |
| RR(times/min) | 20.00 [16.00, 26.00] | 20.00 [16.00, 26.50] | 0.748 |
| Laboratory tests, M (Q₁, Q₃) |  |  |  |
| pH | 7.37 [7.30, 7.43] | 7.38 [7.30, 7.44] | 0.292 |
| lactate(mmol/L) | 2.30 [1.52, 4.00] | 2.20 [1.58, 3.91] | 0.750 |
| AB(mmol/L) | 21.00 [17.17, 24.80] | 20.70 [17.30, 25.05] | 0.737 |
| OI | 214.56 [145.34, 306.82] | 215.15 [155.68, 324.36] | 0.593 |
| BE(mmol/L) | -4.10 [-7.50, -0.24] | -3.90 [-7.90, 0.75] | 0.647 |
| TP(g/L) | 50.70 [45.00, 58.00] | 51.10 [45.70, 60.00] | 0.383 |
| ALB(g/L)(μmol/L) | 27.60 [23.70, 31.50] | 27.90 [23.90, 31.55] | 0.717 |
| TBIL(μmol/L) | 17.30 [11.10, 29.33] | 17.90 [12.15, 27.00] | 0.818 |
| DBIL(μmol/L) | 7.70 [4.20, 14.60] | 7.70 [4.40, 13.45] | 0.984 |
| ALT(U/L) | 27.80 [14.47, 67.25] | 23.00 [14.00, 53.00] | 0.106 |
| AST(U/L) | 46.10 [26.00, 107.95] | 40.70 [23.50, 98.55] | 0.208 |
| K(mmol/L) | 4.04 [3.60, 4.60] | 3.90 [3.53, 4.39] | 0.076 |
| Na(mmol/L) | 139.00 [135.07, 144.00] | 140.00 [136.00, 145.00] | 0.051 |
| Cl(mmol/L) | 105.00 [101.00, 110.00] | 105.70 [101.00, 111.00] | 0.321 |
| Ca(mmol/L) | 1.99 [1.85, 2.16] | 1.98 [1.86, 2.13] | 0.609 |
| PO_4_(mmol/L) | 1.14 [0.82, 1.45] | 1.07 [0.81, 1.40] | 0.564 |
| Mg(mmol/L) | 0.83 [0.71, 0.95] | 0.81 [0.70, 0.93] | 0.239 |
| BUN(mmol/L) | 12.55 [8.10, 20.50] | 11.90 [7.35, 19.20] | 0.147 |
| Cr(μmol/L) | 119.10 [73.22, 186.93] | 100.00 [70.55, 170.50] | 0.169 |
| PCT(ng/mL) | 9.42 [1.52, 43.24] | 11.00 [2.09, 37.33] | 0.525 |
| PT(s) | 14.90 [13.20, 17.00] | 15.10 [13.25, 17.35] | 0.427 |
| PT-INR | 1.33 [1.21, 1.50] | 1.33 [1.21, 1.52] | 0.624 |
| APTT(s) | 34.80 [30.40, 40.85] | 35.00 [30.05, 42.00] | 0.807 |
| FIB(g/L) | 5.50 [3.66, 12.30] | 5.10 [3.60, 7.47] | 0.152 |
| TT(s) | 15.55 [5.73, 17.20] | 15.80 [13.85, 17.15] | 0.356 |
| WBC(10^9^/L) | 12.23 [7.04, 18.16] | 11.54 [7.52, 17.17] | 0.612 |
| NEU(10^9^/L) | 10.84 [5.98, 16.51] | 10.00 [6.44, 15.45] | 0.669 |
| LYM(10^9^/L) | 0.64 [0.36, 1.03] | 0.62 [0.40, 0.99] | 0.815 |
| MON(10^9^/L) | 0.37 [0.18, 0.66] | 0.39 [0.18, 0.62] | 0.95 |
| Hb(g/L) | 105.00 [89.00, 126.00] | 105.00 [88.50, 126.00] | 0.979 |
| RDW(fL) | 48.50 [45.08, 53.82] | 48.50 [45.00, 55.05] | 0.498 |
| PLT(10^9^/L) | 141.00 [85.75, 218.00] | 140.00 [90.00, 230.00] | 0.547 |
| Interventions, n(%) |  |  |  |
| deep venous catheterization | 469 (79.22) | 198 (77.65) | 0.607 |
| anticoagulant drugs | 284 (47.97) | 126 (49.41) | 0.701 |
| mechanical ventilation | 526 (88.85) | 223 (87.45) | 0.559 |
| hormones | 425 (71.79) | 197 (77.25) | 0.099 |
| vasoactive drugs | 566 (95.61) | 240 (94.12) | 0.354 |
| CRRT | 327 (55.24) | 126 (49.41) | 0.119 |
| infusion of human albumin | 433 (73.14) | 191 (74.90) | 0.594 |
| Z: Mann-Whitney test, χ²: Chi-square test | | | |
| M: Median, Q₁: 1st Quartile, Q₃: 3st Quartile | | | |
| APACHE: acute physiology and chronic health evaluation II; SOFA: sequential organ failure assessment; HR: heart rate; SBP: systolic blood pressure; DBP: diastolic blood pressure; MAP: mean arterial pressure; RR: respiratory rate; AB, actual bicarbonate; OI, oxygenation index; BE: base excess; TP: total protein; ALB: albumin; TBIL: total bilirubin; DBIL: direct bilirubin; ALT: alanine aminotransferase; AST: aspartate aminotransferase; K: potassium; Na: sodium; Cl: chloride; Ca: calcium; PO_4_: phosphorus; Mg: magnesium; BUN: blood urea nitrogen; Cr: creatinine; PCT: procalcitonin; PT: prothrombin time; PT-INR: prothrombin time-international normalisation ratio; APTT: activated partial thromboplastin time; FIB: fibrinogen; TT: thrombin time, WBC: white blood cells; NEU: neutrophils; LYM: lymphocytes; MON: monocytes; Hb: hemoglobin; RDW: red blood cell distribution width; PLT: Platelet | | | |

Table S2. Predicted items for validation set

| Variables | SIC (n=84) | Non-SIC (n=66) | *P* |
| --- | --- | --- | --- |
| lactate, M (Q₁, Q₃) | 2.23 [1.47, 3.78] | 1.97 [1.32, 3.09] | 0.106 |
| OI, M (Q₁, Q₃) | 176.12 [114.05, 229.20] | 199.24 [140.52, 277.86] | 0.082 |
| TP, M (Q₁, Q₃) | 50.40 [43.58, 57.65] | 54.20 [49.75, 60.35] | 0.01 |
| TBIL, M (Q₁, Q₃) | 17.10 [12.12, 28.90] | 12.80 [10.00, 22.42] | 0.028 |
| BUN, M (Q₁, Q₃) | 11.46 [6.96, 20.37] | 7.70 [5.58, 11.88] | 0.001 |
| PCT, M (Q₁, Q₃) | 3.04 [0.92, 18.88] | 1.25 [0.15, 10.13] | 0.007 |
| APTT, M (Q₁, Q₃) | 34.45 [29.90, 38.32] | 28.25 [26.20, 30.17] | <0.001 |
| MON, M (Q₁, Q₃) | 0.38 [0.20, 0.59] | 0.36 [0.24, 0.65] | 0.756 |
| SIC: sepsis-induced coagulopathy; OI, oxygenation index; TP: total protein; TBIL: total bilirubin; BUN: blood urea nitrogen; PCT: procalcitonin; APTT: activated partial thromboplastin time; MON: monocytes. | | | |

**Supplementary Figure**

**Figure S1** Patient screening flow in validation set. SIC: sepsis-induced coagulopathy; ICU: intensive care unit.


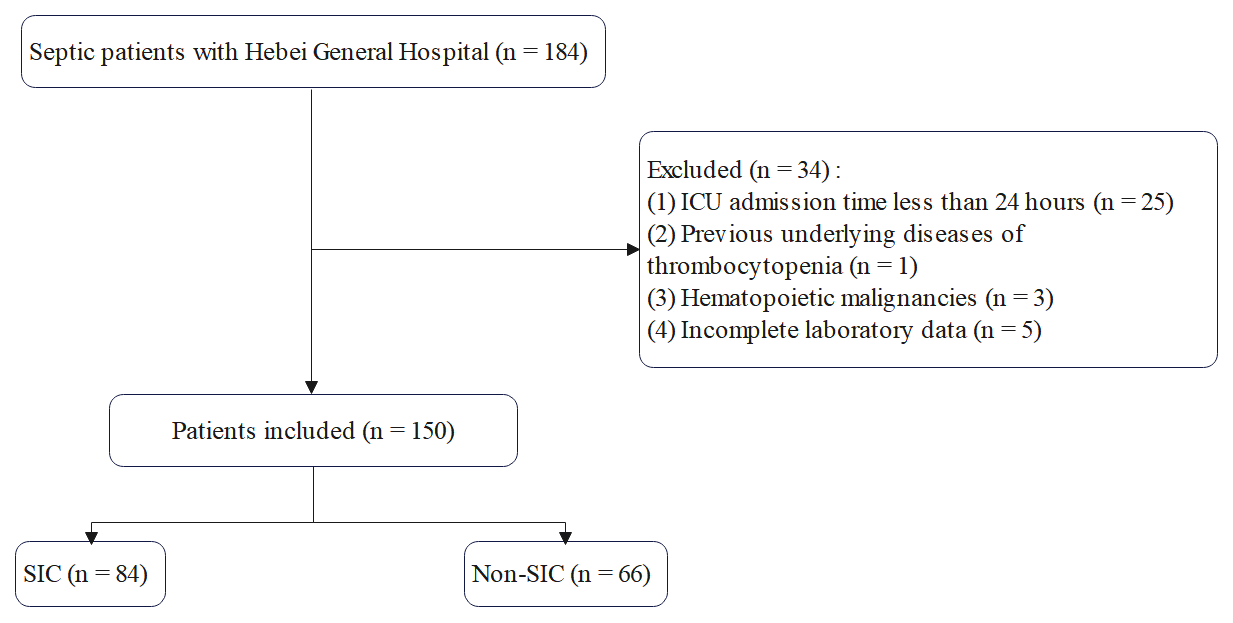


**Figure S2A** ROC curve of the nomogram model in the testing set. ROC: receiver operating characteristic.


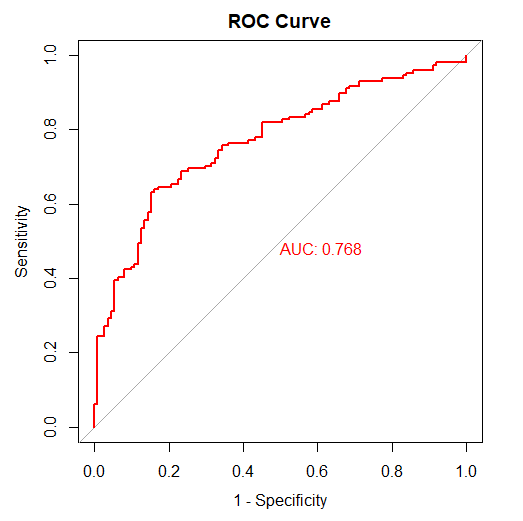


**Figure S2B** ROC curve of the nomogram model in the validation set. ROC: receiver operating characteristic.


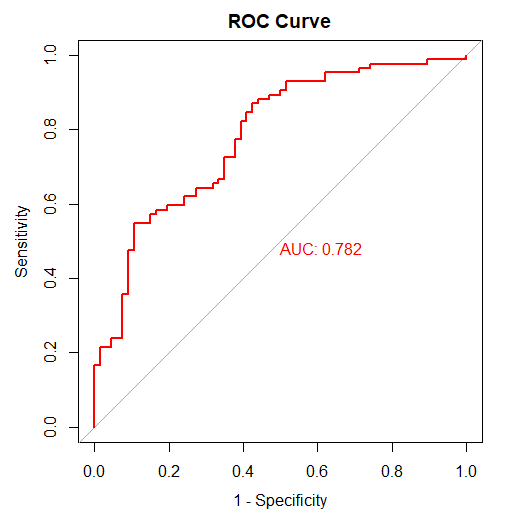


**Figure S3A** Calibration curve of the nomogram model in the testing set.


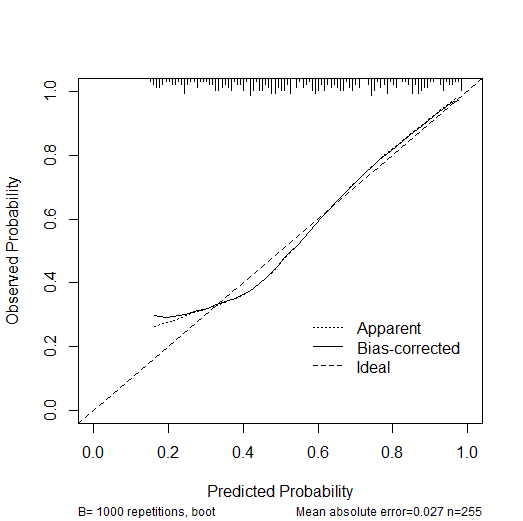


**Figure S3B** Calibration curve of the nomogram model in the validation set.


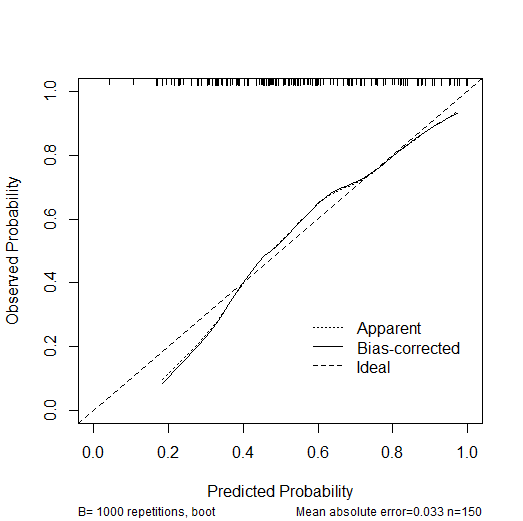


**Figure S4A** DCA curve of the nomogram model in the testing set.


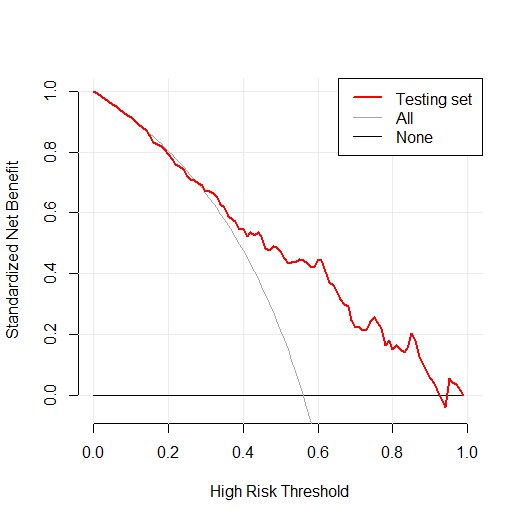


**Figure S4B** DCA curve of the nomogram model in the validation set.


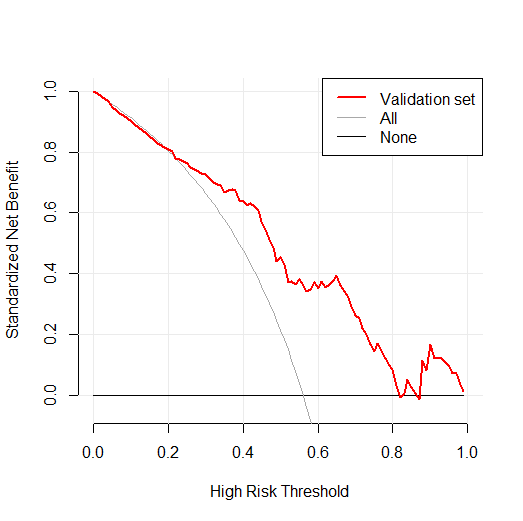

Supplement: Supplementary file 1 [file Data_Sheet_1.docx]
